# Artificial Intelligence Procedures for Tree Taper Estimation within a Complex Vegetation Mosaic in Brazil

**DOI:** 10.1371/journal.pone.0154738

**Published:** 2016-05-17

**Authors:** Matheus Henrique Nunes, Eric Bastos Görgens

**Affiliations:** 1 Forest Ecology and Conservation Group, Department of Plant Sciences, University of Cambridge, Cambridge, CB2 3EA, United Kingdom; 2 Department of Forest Sciences, College of Agriculture, University of São Paulo, Piracicaba, São Paulo, 13418–900, Brazil; 3 Department of Forestry, Universidade Federal dos Vales do Jequitinhonha e Mucuri, Diamantina, MG, 39100–000, Brazil; Universidad Veracruzana, MEXICO

## Abstract

Tree stem form in native tropical forests is very irregular, posing a challenge to establishing taper equations that can accurately predict the diameter at any height along the stem and subsequently merchantable volume. Artificial intelligence approaches can be useful techniques in minimizing estimation errors within complex variations of vegetation. We evaluated the performance of Random Forest^®^ regression tree and Artificial Neural Network procedures in modelling stem taper. Diameters and volume outside bark were compared to a traditional taper-based equation across a tropical Brazilian savanna, a seasonal semi-deciduous forest and a rainforest. Neural network models were found to be more accurate than the traditional taper equation. Random forest showed trends in the residuals from the diameter prediction and provided the least precise and accurate estimations for all forest types. This study provides insights into the superiority of a neural network, which provided advantages regarding the handling of local effects.

## Introduction

Taper models (TM) have been a major topic of study in forest measurement and management for almost 100 years, especially for the past three decades. TM has not been tailored towards understanding the complexity of tropical natural forests, which are among the most structurally complex and carbon-rich ecosystems in the world. This complexity is related to the size-frequency distribution of wood stems [1] and the three-dimensional arrangement of canopy elements, such as leaves, branches and trunks, from the top of the canopy to the ground [2]. Accurate information concerning wood volume in tropical forests is critical in identifying potential areas for sustainable timber production and forest conservation, whilst providing a more accurate estimate of carbon balance. [3] estimated biomass change in buttressed trees using tree taper models, and demonstrated that taper-based equations that are applied to natural forest might improve the modelling of natural forests substantially.

Typical modelling efforts attempt to enhance prediction through amplifying a pattern and discarding the noise. Selection of an appropriate methodology is thus key when performing calculations to estimate biomass accurately. According to [4], volume equations are useful in estimating the average content of standing trees of various sizes and species. However, the reliability of volume estimates is dependent on the range and extent of the available sample data, and the suitability of the volume equations for the given sample data. According to [3], various sources of estimation uncertainty are derived from forest inventories, likely leading to substantially bias in forest biomass and biomass change estimations.

Tropical forests pose a special challenge—because tree taper is dramatically irregular from stump to the top, it is necessary to make some evaluation of stem form in the construction and application of tree volume tables. The rate of tree taper varies not only by species but also by tree age [5], diameter at breast height (*dbh*), height [6] and environmental conditions [7]. In most cases, foresters have to deal with noisy, multi-dimensional data that are strongly non-linear and which does not meet the assumptions of conventional statistical procedures [8]. Artificial intelligence tools have been increasingly adopted over the last 20 years to overcome problems related to lack of statistical assumptions.

Artificial intelligence tools (AI) are capable of handling non-normality, nonlinearity and co-linearity in a system. These capabilities create major advantages for the use of the Artificial Neural Network (NN) as a tool to assess the relationships among structural forest attributes [9]. NN’s provide a particular approach toward developing predictive models, offering a powerful method for analyzing complex relationships among variables, without having to make assumptions about the data. An Artificial Neural Network is an artificial intelligence tool specially designed to deal with complex and ill-defined problems [10]. NN’s can learn from incomplete, disturbed and ‘noisy’ datasets [11].

Another artificial intelligence technique is the Random Forest (RF) tool [12], an ensemble tool that uses a ‘divide-and-conquer’ approach to improving performance. RF constructs hundreds of decision trees (hence ‘forest’) using randomized subsets of predicted and predictor variables [13] These multiple trees are then selected based upon their variation, in order to ascertain the correct prediction [14]. The RF approach has been successfully implemented within the forested ecological system application [13]. [15] indicated RF as the most suitable tool for the classification of various savanna tree species, within a highly heterogeneous environment.

This study aims to evaluate the abilities of Neural Network and Random Forest models in predicting tree diameter (*d*) at any height and any accumulated volume (*Vac*) along the length of stem. This will be accomplished by measuring tree taper across three different sites including a savanna, a dense rainforest, and a semi-deciduous forest. The fitted model predictions will be compared with site-specific taper equation results.

## Materials and Methods

### Data set of the investigation

**Fig 1:**
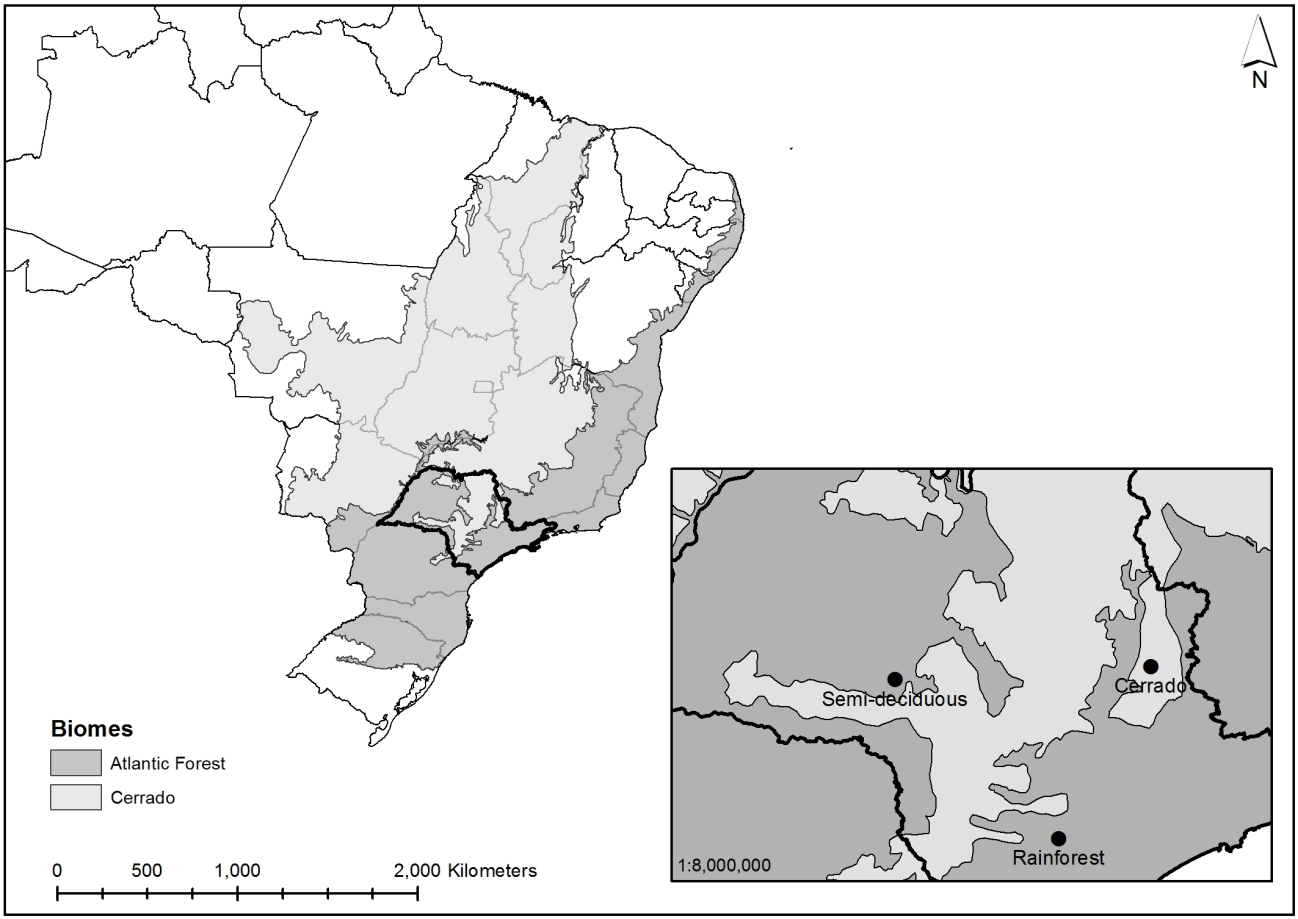
Study Sites. Location of the three study sites in Southeastern Brazil, which are included in two Brazilian biomes (Cerrado and Atlantic Forest).

Fig 1 shows the localities where forest inventories were carried out, including forest type and biome. Mogi Guaçu Biological Reserve belongs to the Instituto de Botânica (22°15’17” S, 47°10’20” W). The reserve is located at an altitude of 620 m, with 343 ha mainly covered by woodland Cerrado; a forested Brazilian tropical savanna. The second area was the north portion of the “Carlos Botelho” State Park (24°03’54” S, 47°57’29” W), at an altitude of 776 m. The total park area consists of 37,797.43 ha of dense ombrophilous montane forest, more common designated as rainforest. The third forest was carried out in the Caetetus Ecological Station (22°24’15” S, 49°41’47” W), at an altitude of 587 m. The vegetation consists of 2,178 ha of tropical seasonal semi-deciduous forest. The transition between coastal rainforest and Cerrado in southeastern Brazil incorporates a much larger extension of semi-deciduous forest. This transition becomes increasingly wider towards the south and forms complex mosaics with Cerrado vegetation to the west [16].

The *Instituto de Botânica* of the State of São Paulo is the regulatory authority issuing work permissions for the Mogi Guaçu Biological Reserve, and the *Instituto Florestal* of the São Paulo State is the authority responsible for issuing work permissions for both Caetetus Ecological Station and “Carlos Botelho” Park Station. We confirm we were given permissions by the two regulatory authorities to conduct this study on the three sites.

The tree vegetation communities were surveyed within thirty plots of 10 x 30 meters each (0.9 ha), with 10 plots in each tree community. In the rainforest and semi-deciduous forest, the sample design followed a random protocol within a buffer zone of 1000 meters along the trails. This protocol had to be used due to the difficulty of the terrain and the denseness of the understory. In the Cerrado, we followed a completely random protocol, distributing the plots randomly within all over the forest area.

Before selecting for volume estimation, we identified trees to species level. The floristic and forest structure held the same characteristics from previous studies carried out on the same sites [17–19]. Subsequently diameter at breast height (*dbh*) outside bark and total height (*ht*) of all of the trees in the plots were measured, and diameter distributions determined to guide tree selection for taper measurements (Table 1).

**Table 1:** Field data summary.

		*ht* (m)		*dbh* (cm)	
	N	Median	Range	Median	Range
Cerrado	531	5.00	1.7–17.0	7.70	5.0–48.4
Rainforest	540	9.65	1.4–26.0	9.34	5.0–107.9
Semi-deciduous forest	446	9.90	2.0–24.0	9.29	5.0–89.1

Diameter at breast height (*dbh*) and total height (*ht*) distributions collected in cerrado, rainforest and semi-deciduous forest in the São Paulo State, Brazil. n = number of measured trees.

**Fig 2:**
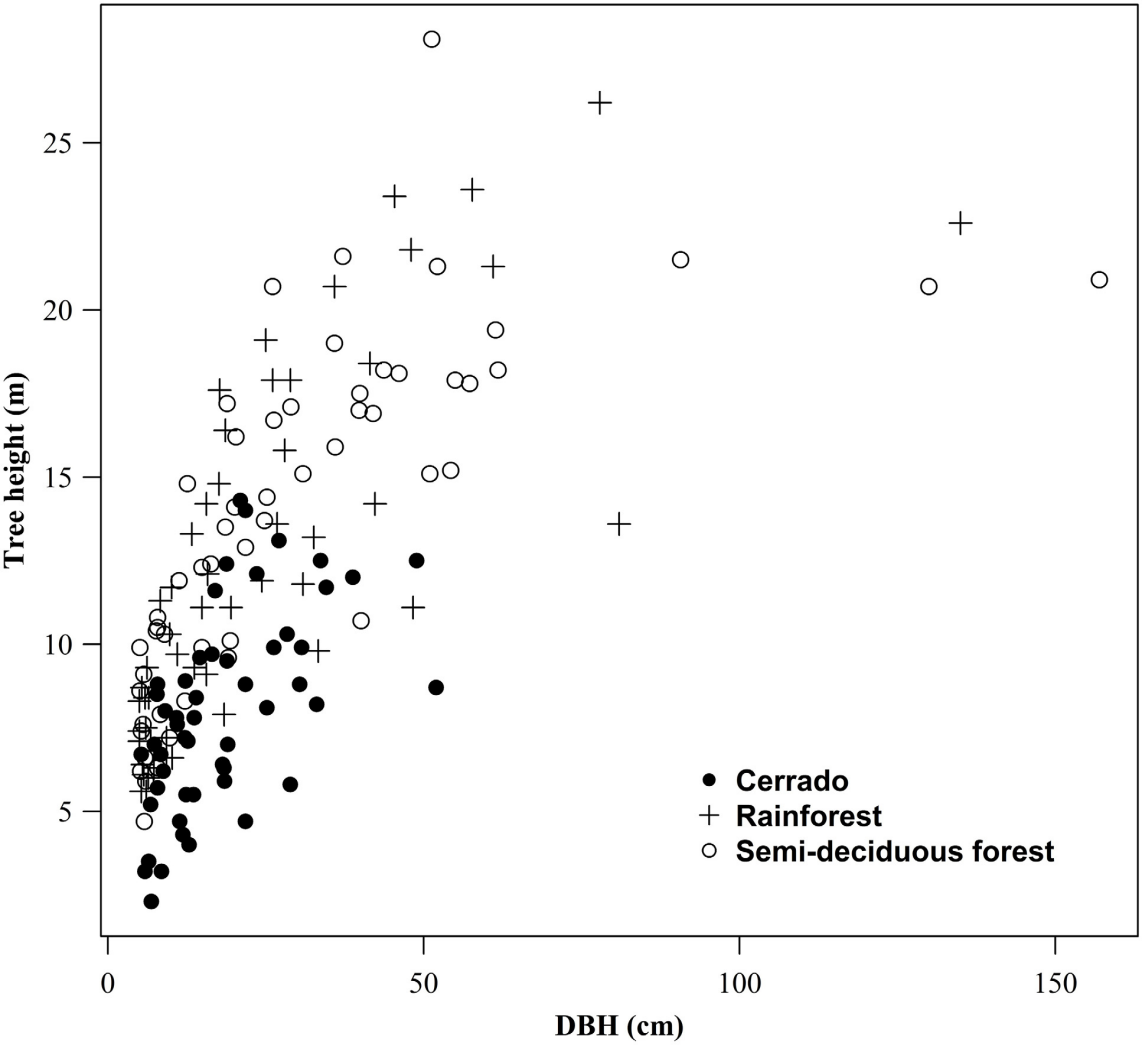
Diameter versus total height scatterplot. Diameter at breast height and total height relationships of trees used as data set in Cerrado, semi-deciduous and rainforest.

We then selected trees from different diameter classes for taper measurements and individual volume estimates regardless of the species. We collected data for taper measurements from 72 hardwood species spread out among all the different forest types, allowing for the fact that individual tree stem forms could vary with the species and forest type. The relationship between tree height and diameter at breast height of the trees selected for taper measurements are shown in Fig 2.

We observed that some species, such as *Couepia grandiflora* and *Qualea grandiflora* in Cerrado, are frequently associated with a complex branching structure, with stems often characterised by thicker diameters. On the other hand, various species in the rainforest, such as *Bathysa australis* and *Alchornea triplinervea*, were usually found to be buttressed and slender. Additionally, we found different species of the genus *Ficus* in the three forest types, which were broadly characterised by large flared stumps (buttressed trees).

Direct volume estimations of different tree parts were made to obtain the basic data underpinning the relationships between the various dimensions of a tree and its volume and taper. The volume outside bark of the stem was calculated using Smalian´s formula, which divides the stem into short sections [20]. Measurements included the portion of the stem above 10 cm height and then at 0.3, 0.7, 1.3 meters. From 1.3 m up to a minimum of 5 cm stem diameter from the outer edge of the bark, the stem was measured at intervals of 1 meter. In order to avoid problems with discerning the main stem, we measured all branches of trees with a minimum 5 cm diameter. Above the final measurement point, the tree form was considered as a cone. We followed the recommendation of [21] concerning multi-stemmed trees, whereby all of the stems should be measured and combined with the equivalent diameter formula below:

$$D_{e}=\;\sqrt{\left(d_{1}^{2}+\;d_{2}^{2}\;+\;\ldots \;+\;d_{n}^{2}\right)}$$ (1)

*D*_*e*_ = equivalent diameter and *d*_*i*_ = diameter of a specific stem i = 1,…,n from a single tree.

We used the electronic dendrometer Criterion RD 1000 (Laser Technology, Inc., USA) to measure stem diameter. It is an optical instrument that provides real-time results for tree height and diameter calculation along the stem with high accuracy[22]. [23] did not detect significant differences in precision and accuracy between destructive measurement techniques and the Criterion RD 1000. The dendrometer uses angular measurement and horizontal distance to the target tree in order to calculate the diameter of the tree stem at any given height.

The advantage of this definition is that nearly all potentially useful wood is included. Total tree volume estimated using equivalent diameters is equal to totalling the estimates of individual stem volume in a multi-stem tree. Using equivalent diameter also permits calculating the real tree basal area which can be used as a predictor of individual tree volume. Relationships between tree basal area and cubic volume are stronger than relationships between tree basal area and merchantable volume such as board foot volume.

### Artificial Neural Network

Two multi-layer perceptron (NN) were calibrated in the context of regression analyses, one to estimate the diameter (*d*) and another to estimate the accumulated volume (*Vac*) from the base up to a given height (*h*). Both contained two hidden layers: 25 neurons in the first and 10 neurons in the second, all containing the logistic as the activation function. The NN training was oriented to minimize the sum of squared errors through resilient backpropagation algorithm with weight backtracking. For each iteration of the cross-validation the NN was initialized 50 times, and the training ended when the absolute partial derivative of the error function, with respect to the weights, was smaller than 0.01. Similar to regular taper equations, we used NN to estimate either diameter or accumulated volume at *h* based on *dbh*, *ht* and *h*. However, these variables were scaled before NN analysis by dividing them, respectively, by 100, 10 and 10. Besides the continuous variables *dbh*, *ht* and *h*, the NN also received as input three dummy (categorical) variables representing the forest type. In Cerrado the dummy variable 1 (*d1*) received value 1, while the other forest types received value 0. In semi-deciduous the dummy variable 2 (*d2*) received value 1 and the others received value 0. In rainforest the dummy variable 3 (*d3*) received value 1, while cerrado and semideciduous received value 0. For instance, in order to estimate either *d* or *Vac* for a given height equal to 4.3 meters in a tree from Cerrado, with *dbh* equal to 53 cm and 8.7 meters in height, the input vector should be [0.53, 0.87, 0.43, 1, 0, 0]. The implementation of the neural network was based on the *neuralnet* package [24] for R statistical software [25].

### Random Forest

Two random forests (RF) were used, one to estimate *Vac* and another to estimate *d*. The RF inputs include *dbh* in cm, *ht* and *h*, both in meters, as well as three dummy variables indicating the forest type (Cerrado = *d1*, semi-deciduous = *d2* or rainforests = *d3*). The RF was implemented through the algorithm developed by [12], and built using 300 decision-trees, m_try_ (randomly sampling from the predictors) equal to 2 and the minimum observation per node equal to 5 after split. The objective of the training section was to minimize the sum of squared errors. We built the RF models using the R package *randomForest* [26].

The parameters mentioned for NN and RF were selected by a trial-and-error method, testing a range of possible values and then verifying the graphs of residuals against the predicted variables and fitting statistics. Trial-and-error method is commonly used to define parameters in the field of artificial intelligence [27].

### Taper model

We selected 6 taper models proposed in the literature with different number of parameters that had previously shown good performance (Table 2) [28–33]. The taper equations were adjusted using nonlinear least-squares estimates through a Gauss-Newton algorithm, implemented in *stats* package in R [25] and then we compared the goodness-of-fits using the Akaike Information Criterion corrected for finite sample sizes (AICc). We determined the best overall taper model by counting the number of times that each model provided the lowest AICc for the three forest types.

**Table 2:** Taper equations.

Demaerschalk [28]	$d\;=\;dbh\sqrt{\left(10^{2{*\beta }_{0}}\text{*}dbh^{2{*\beta }_{1}-\;2}\text{*}ht^{2{*\beta }_{2}}\text{*}{\left(ht-h\right)}^{2{*\beta }_{3}}\;\right)}$
Biging [29]	$d=\;dbh\text{*}\left\{\beta_{0}+\beta_{1}\text{ln}\left[1-{\left(\frac{h}{ht}\right)}^{\frac{1}{3}}\text{*}\left(1-{\text{exp}}^{\frac{-\beta_{1}}{\beta_{2}}}\right)\;\right]\right\}$
Bi [30]	$d=dbh\text{*}\left\{{\left[\frac{\text{ln sin}\frac{\pi }{2}q}{\text{ln sin}\frac{\pi }{2}t}\right]}^{\beta_{1}+\beta_{2}\text{sin}\frac{\pi }{2}q\;+3\text{cos}\frac{3\pi }{2}q+\beta_{4}\frac{\text{sin}\frac{\pi }{2}q}{q}+\beta_{5}dbh+\beta_{6}q\sqrt{dbh}+\beta_{7}q\sqrt{ht}\;}\right\}$
Lee et al. [31]	$d\;=\beta_{1\;}dbh^{\beta_{2\;}}{\left(1-q\right)}^{\beta_{3\;}q^{2}+\beta_{4\;}q+\beta_{5\;}}$
Kozak [32]	$d=\beta_{1\;}dbh^{\beta_{2\;}}\;ht^{\beta_{3\;}}X^{\beta_{4}q^{4}+\beta_{5}\left(\frac{1}{e^{dbh/ht}}\right)\;+\beta_{6}X^{0.1}+\beta_{7}\left(\frac{1}{dbh}\right)+\beta_{8}ht^{Z}+\beta_{9}X}$
Metcalf et al. [33]	$d\;=\;D\;{\text{e}}^{-\beta_{1}\;\left(h-1.3\right)}$

Taper equations compared in this study, where *dbh* is the over bark diameter at breast height (at 1.3 m above the top of the base, cm), *d* is the over bark diameter at height *h* (cm), *ht* is the total tree height (m), *h* is the height from the base to diameter *d* (m), β_1_,…,β_9_ are the model parameters to be estimated, *q* is equal to *h/ht*, *X* is equal to [1 − (*h*/*ht*)^1/3^]/[1 − (*p*)^1/3^], *Z* is equal to [1 − (*h*/*ht*)^1/3^] and *p* is equal to 1.3/*ht*.

Integrating taper functions over the length desired in meters gives the volume in cubic meters for that segment, after multiplying by a constant (K = π⁄40,000).

$$Vac=\int_{0.1}^{h}\text{K*}d^{2}\;d_{h}$$ (2)

As the tree volume is the integral of cross-sectional stem area over the tree height, a model for *d*^2^ provides unbiased predictions for the cross-sectional area and volume [34]. The category of the taper model we used is very flexible in a computational sense, since it is possible to determine the continuous stem taper with the model itself and no interpolation method, such as spline interpolation, is needed [35]. We also did not consider eventual autocorrelation and multicollinearity effects in this paper, as [36] evaluating these problems on tree taper modelling stated that they do not seriously affect the predictive ability of taper modelling. One specific equation had to be adjusted for each study site, consequently returning a site-specific model (one taper model to Cerrado, one to semi-deciduous and one to rainforest), while the RF and NN modelling processes considered all the forest types together.

#### Evaluation criteria

For Neural Network (NN), Random Forest (RF) and taper equation modelling (TM), the cross-validation approach was used as training routine, including a tolerance limit to avoid overfitting. The cross-validation by itself does not avoid overfitting but allowed us to understand how the model behaves whilst estimating known and unknown data. For all the three proposed techniques, the data were divided into training and validation datasets. For that, we set aside randomly 25% of the trees for cross-validation purposes, while 75% of the data remained as training dataset for fitting the models. The data splitting was repeated 500 times (iterations) with repetition of the training and the validation steps.

In each iteration, the performance indicators were calculated for both training and validation datasets. Evaluation criteria included the root mean squared error Eq (3), the average relative bias Eq (4) and the model efficiency Eq (5).

$$-\text{ Root mean square error }\left(\text{RMSE}\right)=\;\frac{1}{N}\;\sqrt{\sum \sum {\left({\hat{\;Y}}_{ij}-Y_{ij}\right)}^{2}}\;$$ (3)

$$-\text{ Relative average bias }\left(\text{Bias}\right)\;=\;\frac{\sum \sum \left({\hat{Y}}_{ij}\;-\;Y_{ij}\right)}{\text{N}}$$ (4)

$$-\text{ Efficiency }\left(\text{EF}\right)=\;1-\frac{\sum \sum {\left(Y_{ij}-{\hat{\;Y}}_{ij}\right)}^{2}}{\sum \sum {\left(Y_{ij}-\bar{Y}\right)}^{2}}\;$$ (5)

where *Y*_*ij*_ is the measured data point jth in the ith tree, ${\hat{Y}}_{i}$ is the predicted value jth in the ith tree and $\bar{Y}$ is the mean of the *Y*_*ij*_ values and *N* the number of points. For detailed descriptions of model evaluation criteria see [37].

## Results

The number of trees surveyed resulted in 52 individuals in the Cerrado, 53 in the semi-deciduous forest and 55 in the rainforest, in different diameter classes. The diameter ranged between 5.0 to 52.0 cm in Cerrado, 5.0 to 135.0 cm in semi-deciduous and 5.1 to 157.0 in rainforest.

The TM selected in this study was proposed by [31] with the lowest AICc in semi-deciduous forest and rainforest (Table 3). We used initial parameters based upon the literature to find convergence, however we found no convergence by using Bi model for the three forest types and Kozak model for semi-deciduous and rainforest.

**Table 3:** Taper models performance.

Taper Model	Cerrado	Semi-deciduous	Rainforest
Demaerschalk [28]	2530.21	3530.21	4731.91
Biging [29]	2607.78	3550.15	4715.24
Bi [30]	No convergence	No convergence	No convergence
Lee [31]	2546.00	**3462.49**	**4391.26**
Kozak [32]	**2474.62**	No convergence	No convergence
Metcalf [33]	2829.13	3970.84	4905.55

Model fit comparisons in terms of AICc for 6 taper models. Bold values indicate the lowest AICc for each forest type

Table 4 summarizes the RMSE and model efficiency estimates of the NN, the RF and the TM for *d* and *Vac* estimations from both training and validation datasets.

**Table 4:** Modelling techniques performance.

	Diameter (*d*)		Accumulated volume (*Vac*)	
Approach	RMSE (cm): training / validation	EF: training / validation	RMSE (m³): training / validation	EF: training / validation
**NN**	0.15 / 0.43	0.93 / 0.83	0.0037 / 0.0149	0.98 / 0.89
**RF**	0.18 / 0.50	0.91 / 0.78	0.0082 / 0.0281	0.92 / 0.66
**TM**	0.15 / 0.31	0.94 / 0.91	0.0080 / 0.0225	0.93 / 0.80

The average root mean square error (RMSE) and model efficiency (EF) for diameter (*d*) and accumulated volume (*Vac*) prediction within all forest types using Neural Network (NN), Random Forest (RF) and taper model (TM).

The validation results showed that the site-specific taper equation was the most precise and efficient modelling technique for diameter estimation, with a RMSE of 0.31 cm for TM, 0.43 cm for NN and 0.50 cm for RF. The TM training efficiency declined from 0.94 to 0.91 at the validation level, whilst both NN and RF efficiency declined more than the TM, varying from 0.93 to 0.83 and 0.91 to 0.78, respectively.

**Fig 3:**
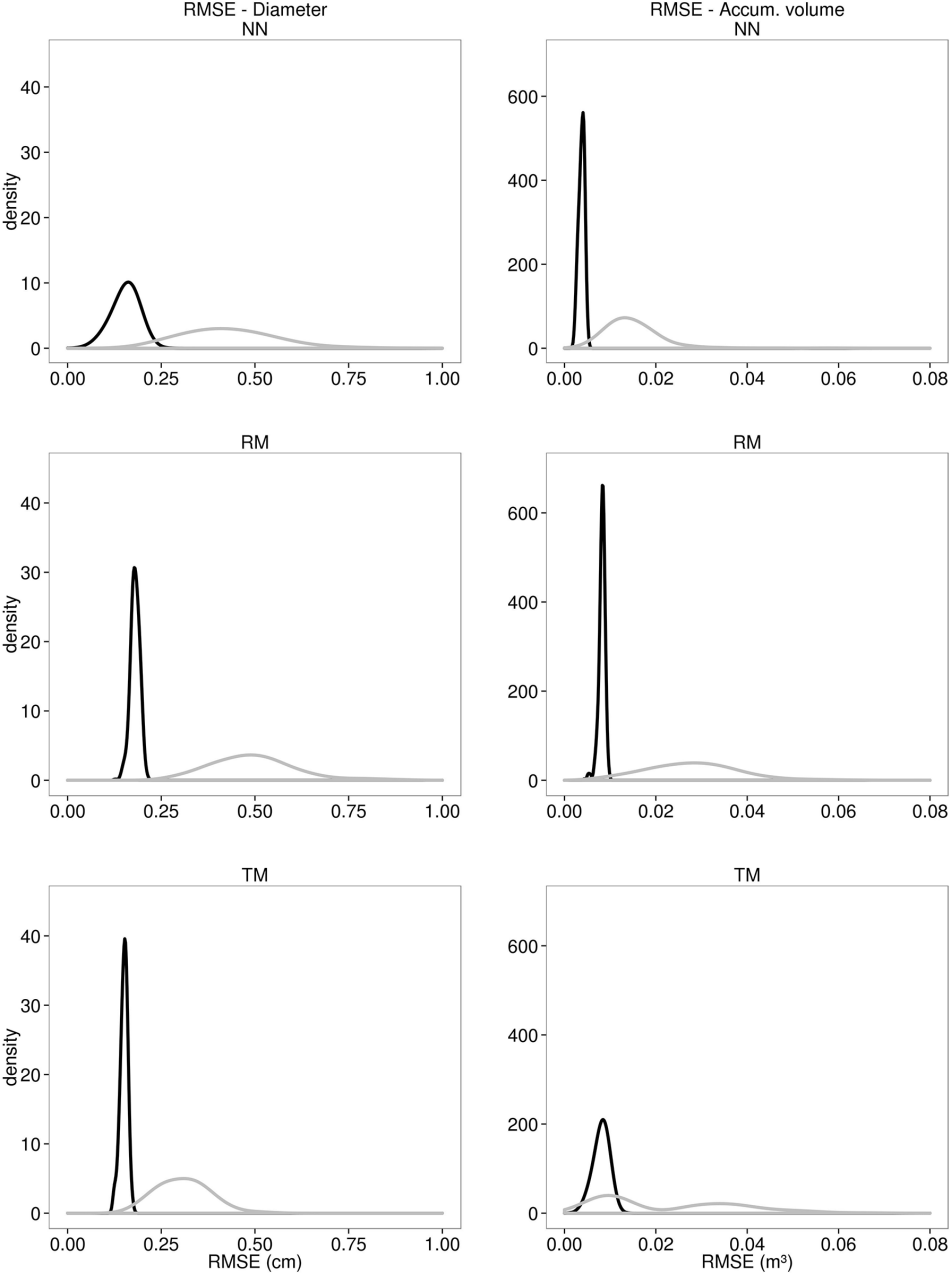
Root mean square error. The root mean squared error (RMSE) distribution of diameter (*d*) and accumulated volume (*Vac*) for both training (black line) and validation (grey line) data sets, considering five hundred iterations for Artificial Neural Network (NN), Random Forest (RF) and taper model (TM).

**Fig 4:**
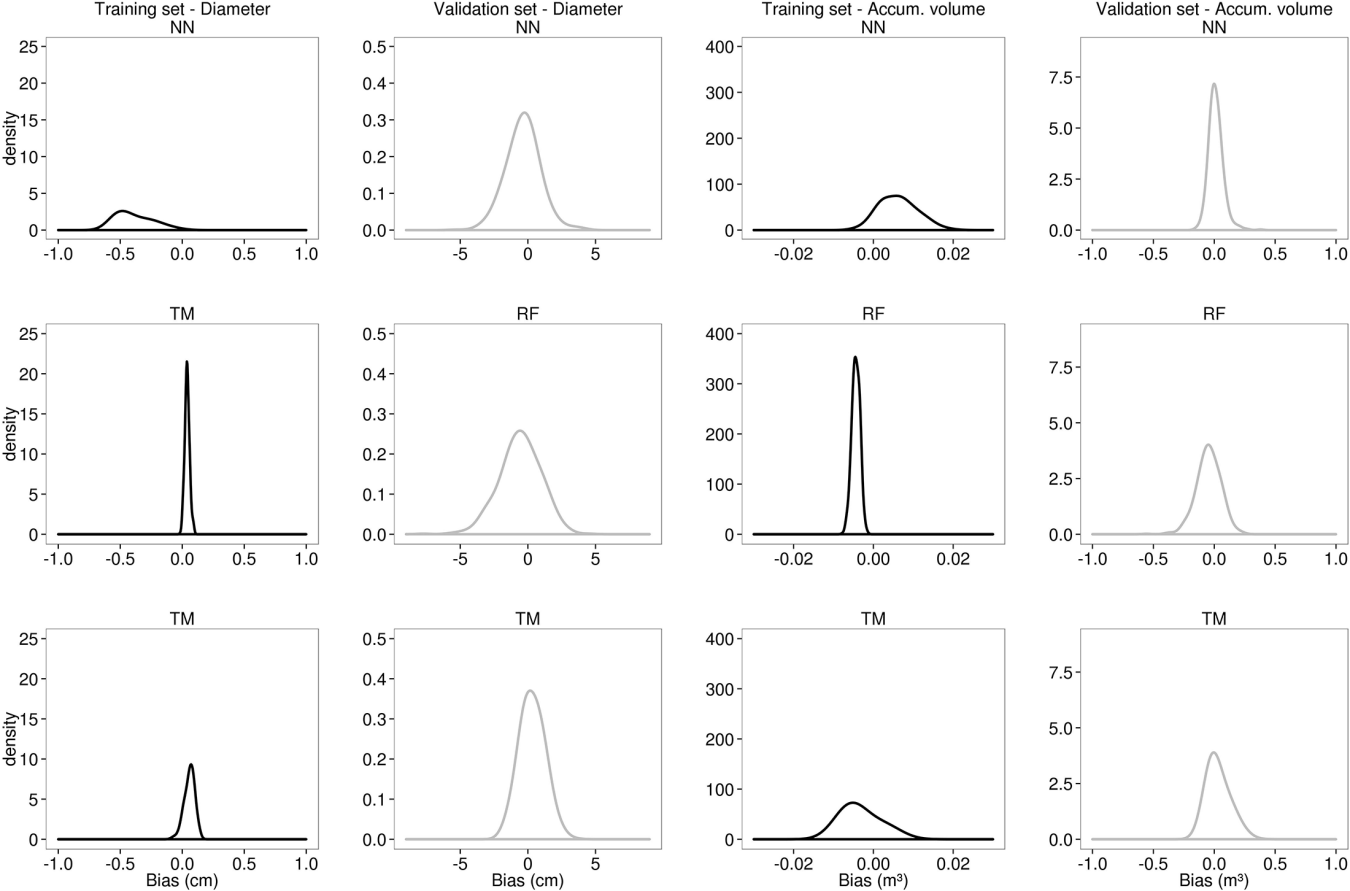
Bias distribution. The bias distribution of diameter (*d*) and accumulated volume (*Vac*) for both training (black line) and validation (grey line) data sets, considering five hundred iterations for Artificial Neural Network (NN), Random Forest (RF) and taper model (TM).

TM did not show the same performance for *Vac* estimation, whilst NN appeared to have the best performance and the higher efficiency. The RF has also presented the worst performance for volume estimation for all the evaluated criteria. Although the TM showed an intermediate RMSE (0.0225 m³), its distribution had an undesirable bimodal shape, ranging approximately from 0 to 250% (Fig 3). All the three methods showed a skewed bias distribution during the training level for both *d* and *Vac*, especially the NN. However, the bias distribution in the validation level did not show the same tendency, appearing centred on zero for all the three techniques (Fig 4).

**Fig 5:**
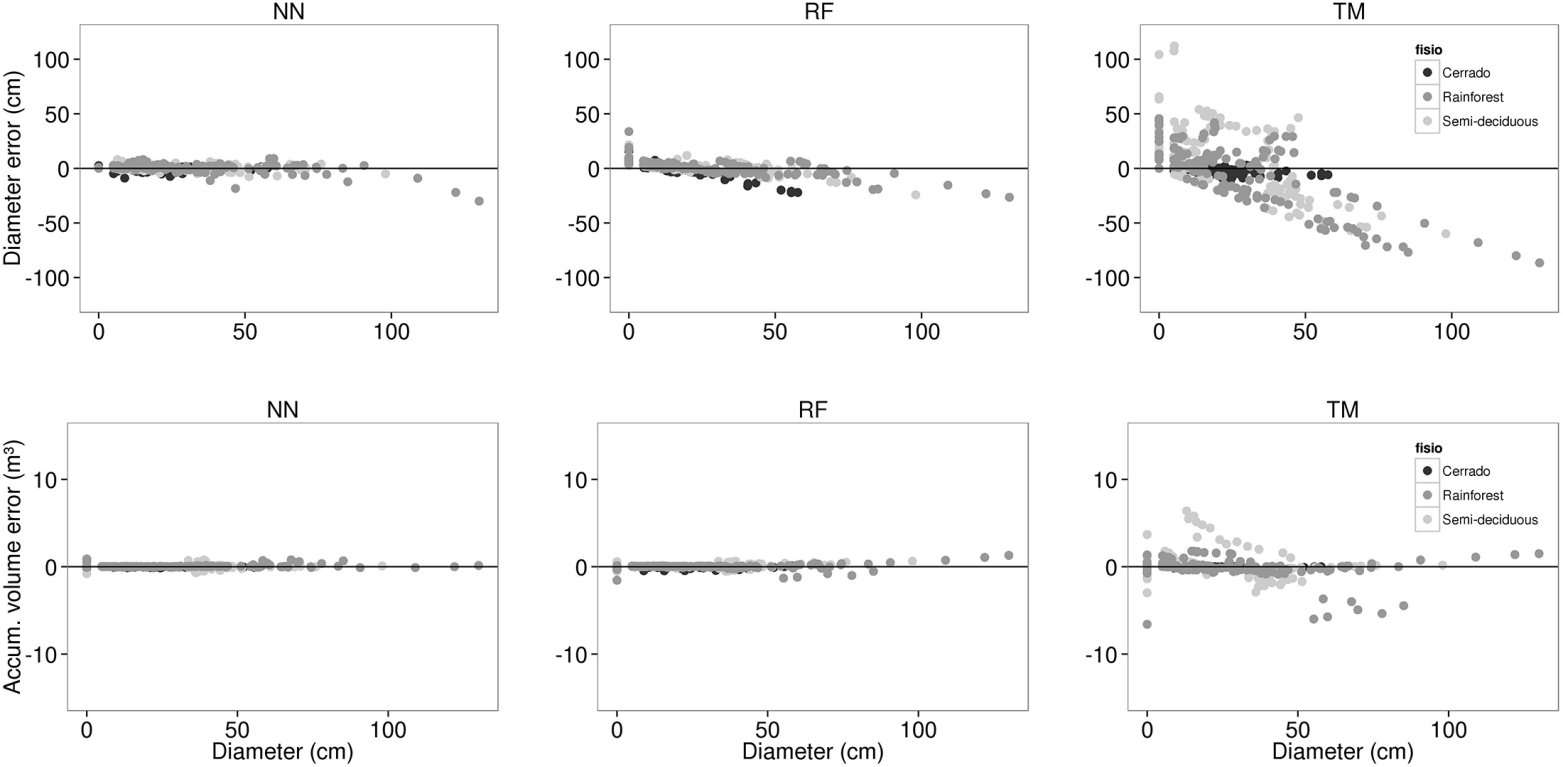
Diameter predictions residuals. Residuals of diameter predictions (cm) versus tree stem diameters (cm) in the upper plots, as well as residuals of accumulated volume predictions (m³) versus tree stem diameters (cm) in the lower plots. Residuals were calculated using the model with the lowest RMSE along 500 iterations using Artificial Neural Network (NN), Random Forest (RF) and taper model (TM) techniques.

We plotted the residuals of *d* and *Vac* predictions versus diameter (*d*) for Cerrado, semi-deciduous forest and rainforest (Fig 5). Residuals were calculated using the model with the lowest RMSE along 500 iterations for the three modelling techniques. RF and TM showed residual patterns that reveal likely variance heterogeneity in diameter estimation. They tended to underpredict large diameters, which are typically associated with diameters on lower and thicker portions of the stem or diameters of large trees. TM and RF also tended to overpredict small diameters, which are related to diameters of smaller trees or diameters on the midrange or upper portions of the stem.

**Fig 6:**
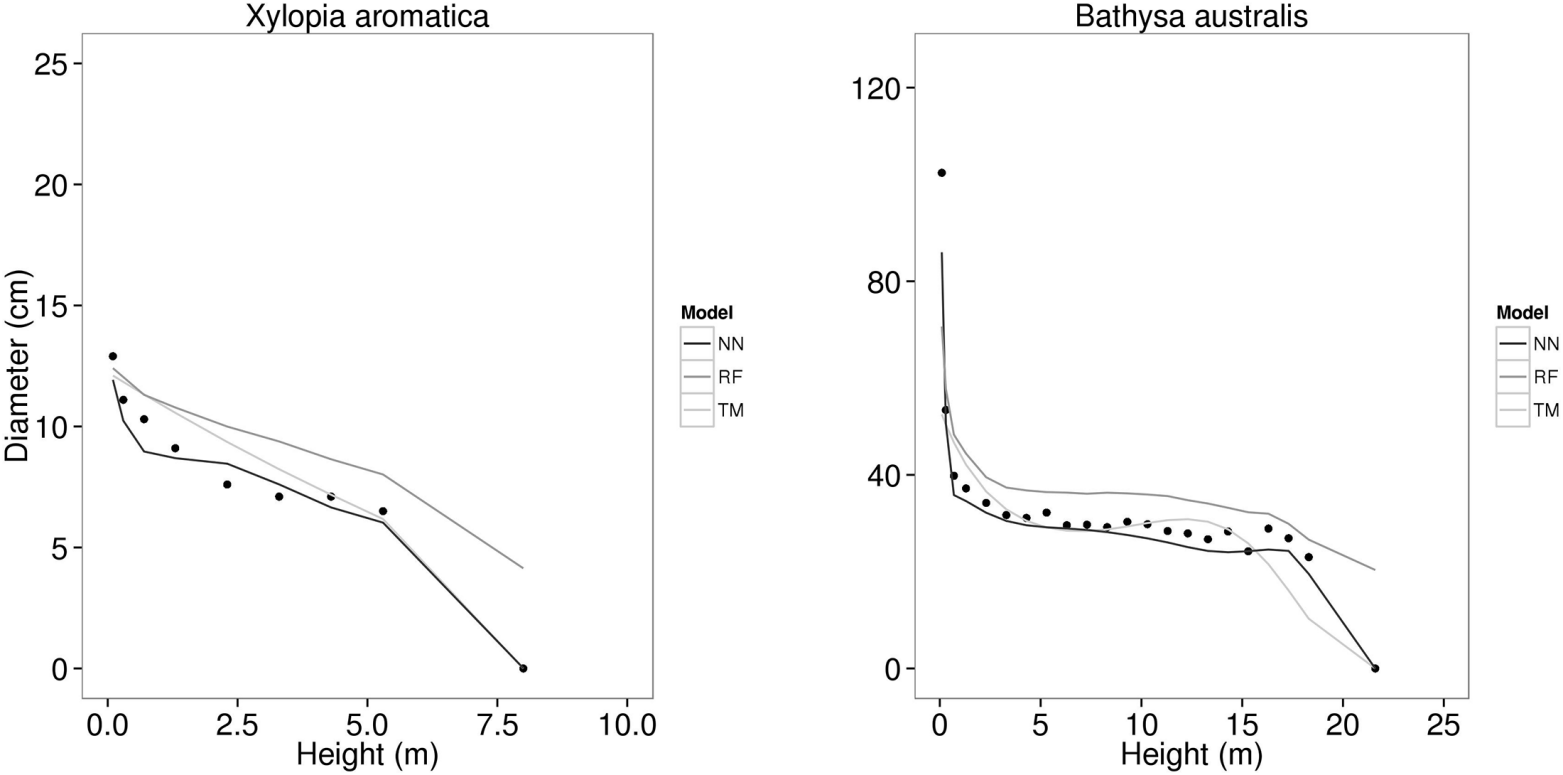
Stem taper predictions. Measured diameters (black dots) of trees with regular (*Xylopia aromatica*) and irregular (*Bathysa australis*) stem taper with the respective predictions fitted by Neural Network (NN), Random Forest (RF) and taper model (TM).

Unlike the residual plots for diameter estimation, we observed no pattern in residuals of volume prediction for any method used to modelling stem taper. Nevertheless, NN plots visually seem to lead to more accurate and precise volume estimation at any diameter class in comparison to the TM and RF. We randomly selected one tree from a group of species which has consistent stem taper and one individual from a group which includes highly irregular stem. The best model for each modelling technique based upon the RMSE predicted diameters along both trees and predictions were compared to actual diameters (Fig 6). *Xylopia aromatica* is a tree species in Cerrado which has a simple and rectilinear stem form, whilst *Bathysa australis* is commonly found in the rainforest with a complex and buttressed stem. The NN technique was more consistent with the actual taper for both species whilst RF tended to overpredict the diameter on both stems.

## Discussion

Our objective was to modelling the stem form as a dependent variable upon the diameter at breast height and the total tree height in different forest types. Taper variation differed according to species composition and tree size; nonetheless several other factors that were not examined here would also influence this variation on stem form. Trees get increasingly more cylindrical as they grow, and dominant individuals are more tapered than suppressed trees. It indicates the likely dependence of taper upon the variables of stand density and tree [38]. Genetics, environmental conditions, which include climatic conditions [39,40], altitude [40] and edaphic variables [41], as well as geographical locations were also listed as factors that can affect the stem taper [42, 43]. Tree stem typically varies according to conditions of the forest, usually as a response to surrounding species and competition. In this cases, individuals are forced to develop more complex structures gradually in order to optimise biomass production [7]. Because of these many sources of variations on stem form, establishing efficient methods that can provide accurate estimates of stem taper is often a challenging process in natural tropical forests.

Random Forest appears as a competitive tool in ecological applications for both classification and regression [44]. However, the least accurate results for diameter and wood volume were obtained by using RF. This model tended to overpredict low diameter and underpredict high diameter values. This particular trend is intrinsic to regression tree-based models whose predictions are the average of the values within the terminal node [45]. These authors also observed a reduction in the prediction accuracy when testing an independent set of data with RF in an effort to estimate biomass across tropical Africa. [46], when studying climatic and human influences on fire regime in Africa, also found overprediction in lower classes and underprediction in higher classes of burned areas.

Very few studies have used taper functions for profile modelling in either Cerrado or Atlantic forests in Brazil. Few of them attempt to describe the stem form and estimate taper-equation parameters for overall stands [47,48], or for specific species [49]. TM provided a flexible tool for estimating the change in total and merchantable product specifications, even though this regression technique requires a specific model for each different forest type. In comparison, the NN and RF techniques required only one model for all three datasets. One problem found in this study regarding the traditional taper modelling is the lack of convergence of parameters in more complex models, which was previously addressed in other studies on taper [50–52].

Given the difficulty in separating out the influences of the stem form drivers using standard statistical analyses, NN appears to be a promising approach for complex vegetation mosaics. It included uneven-aged multi-stemmed, buttressed, sinuous and slender trees and shrubs, varying substantially within forests where the inventories were carried out. Another interesting NN property is that all the knowledge is stored in the weights. If new trees become available, the training can occur on the weights already known keeping all the knowledge accumulated from previous data sets.

[53] verified poor results when using NN for estimating tree height with diameter as the input variable in uneven-aged forests. Considering that these forest stands consist of trees of various ages and therefore of various sizes, each diameter class is consequently associated with a likely height class. However, those authors suggest that the diversity in stem form derived from multi-site variables may hinder the learning, due to each diameter class that may be associated with a larger height class. Backpropagated errors in this scenario are, therefore, larger and the fitting statistics poorer. In this particular study, we attempted to predict stem form based on the highly dependent height and diameter [54]. Small backpropagated errors are expected due to the high correlation between independent and dependent variables.

Studies have demonstrated the superiority of NN’s over regression models for even-aged forests [55–58]. NN offers some advantages when compared to traditional modelling techniques. Firstly, there is no need to assume an underlying data distribution (as is usually done in statistical modelling). Secondly, it can implicitly detect complex nonlinear relationships between output and input variables [59]. Furthermore, the ability to learn from new data allows for continued implementation in situations where only limited amounts of data have been collected [60]. It is important to mention, however, some barriers to the widespread successful application of artificial intelligence. AI demands much training time and can easily incur data overfitting [59, 61]. Another serious limitation is that the most important decision support systems in forestry are not yet able to handle with AI. Moreover, whilst visible, the process of establishing causation between inputs and outputs is not clear, implying limited ecological interpretability [62, 58].

The NN implementation does offer a number of advantages for taper prediction in tropical forests over the traditional methods. It may be potentially applied to large geographic regions in Brazil, handling local effects concerning timber inventory and forest management plans. Furthermore, AI can be continuously trained as new data are obtained and disposable. These statistical considerations discussed above should be taken into account when choosing a tree taper estimating method for operational applications.

## Conclusions

The neural network handled well with data from three different forest types within a complex vegetation mosaic in Brazil. Additionally, the neural network procedure provided an understanding of the patterns that arise from complex phenomena, insofar as correctly training the model and performing prediction. Thereby we recommend NN for taper and volume predictions in tropical forests, especially when stem form and variation in tree architecture is complex. However, our recommendation must be followed by an effort to integrate artificial intelligence tools into current forestry support decision systems.

## Supporting Information

S1 File Brazilian taper data. Comma separated value file with the measured trees.
(CSV)

S2 File RData. Adjusted models for each technique: Random Forest, Neural Network and Taper Models.
(ZIP)
